# In Silico Structural and Functional Characterization of the RSUME Splice Variants

**DOI:** 10.1371/journal.pone.0057795

**Published:** 2013-02-28

**Authors:** Juan Gerez, Mariana Fuertes, Lucas Tedesco, Susana Silberstein, Gustavo Sevlever, Marcelo Paez-Pereda, Florian Holsboer, Adrián G. Turjanski, Eduardo Arzt

**Affiliations:** 1 Instituto de Investigación en Biomedicina de Buenos Aires (IBioBA)-CONICET- Partner Institute of the Max Planck Society, Buenos Aires, Argentina; 2 Departamento de Fisiología y Biología Molecular, FCEN, Universidad de Buenos Aires, Buenos Aires, Argentina; 3 Departamento de Neuropatología y Biología Molecular, FLENI, Buenos Aires, Argentina; 4 Max Planck Institute of Psychiatry, Munich, Germany; 5 Laboratorio de Bioinformática Estructural, Departamento de Química Biológica, INQUIMAE-CONICET, FCEN, Universidad de Buenos Aires, Buenos Aires, Argentina; National Centre for Cell Science, India

## Abstract

RSUME (RWD-containing SUMO Enhancer) is a small protein that increases SUMO conjugation to proteins. To date, four splice variants that codify three RSUME isoforms have been described, which differ in their C-terminal end. Comparing the structure of the RSUME isoforms we found that, in addition to the previously described RWD domain in the N-terminal, all these RSUME variants also contain an intermediate domain. Only the longest RSUME isoform presents a C-terminal domain that is absent in the others. Given these differences, we used the shortest and longest RSUME variants for comparative studies. We found that the C-terminal domain is dispensable for the SUMO-conjugation enhancer properties of RSUME. We also demonstrate that these two RSUME variants are equally induced by hypoxia. The NF-κB signaling pathway is inhibited and the HIF-1 pathway is increased more efficiently by the longest RSUME, by means of a greater physical interaction of RSUME267 with the target proteins. In addition, the mRNA and protein levels of these isoforms differ in human glioma samples; while the shortest RSUME isoform is expressed in all the tumors analyzed, the longest variant is expressed in most but not all of them. The results presented here show a degree of redundancy of the RSUME variants on the SUMO pathway. However, the increased inhibition conferred by RSUME267 over the NF-κB signaling pathway, the increased activation over the HIF-1 pathway and the different expression of the RSUME isoforms suggest specific roles for each RSUME isoform which may be relevant in certain types of brain tumors that express RSUME, like human pituitary adenomas and gliomas.

## Introduction

RSUME is a small protein whose gene was isolated by differential expression screening using a tumor cell line with an increased tumorigenic and angiogenic potential [1], [2]. To date, four human RSUME mRNA splice variants have been described. Three of them mainly differ in their 3′ coding region and 3′ UTR, and codify for three different RSUME isoforms; while the shortest RSUME isoform is composed of 195 aminoacids, the longest RSUME variant has 267 residues, and the third isoform is 200 residues long. We termed this isoforms RSUME267, RSUME195 and RSUME200. The fourth splice variant is a non-coding RNA that suffers nonsense-mediated RNA decay (NMD) [3], a mechanism of degradation of mRNA with premature termination codons.

We have previously shown that RSUME195 increases protein SUMOylation, a dynamic post-translational modification of proteins [4], [5], by interacting physically with the SUMO E2 conjugase Ubc9 [1]. Moreover, RSUME195 increases noncovalent binding of SUMO1 to Ubc9 and consequently enhances Ubc9-SUMO thioester formation [1]. Importantly, the SUMO enhancer properties of RSUME195 depend on the integrity of its RWD domain [1]. This domain was originally identified in three major RWD-containing proteins: RING finger-containing proteins, WD-repeat-containing proteins, and yeast DEAD (DEXD)-like helicases [6]. Several RWD-domain containing proteins have been described to date, and the solution structure of most of them has been determined, suggesting it is a protein-protein interaction domain [6]. Importantly, despite the apparent absence of sequence similarity, the RWD structure significantly resembles that of ubiquitin-conjugating enzymes (E2s) [7]. Indeed, it has been demonstrated that the RWD domain is structurally homologous to the E2-SUMO conjugase Ubc9 [7], [8]. Thereby, a structural homology between RSUME and Ubc9 has been proposed [1]. The RWD domain encompasses aminoacids 1 to 128 of RSUME, and considering that all RSUME variants share their first 176 residues, the RWD domain is contained in all of them.

In previous works it has been shown that RSUME195 is induced by low oxygen levels via a mechanism involving Hypoxia Inducible Factor-1 (HIF-1) [1], [9], the main transcription factor of the adaptive response to hypoxia [10], [11]. HIF-1 induces RSUME195 via the functional HRE (Hypoxia Responsive Element) in the RSUME promoter [1], [12]. Furthermore, we showed that RSUME195 increases the protein levels of HIF-1alpha, the oxygen-regulated subunit of HIF-1 [1]. By increasing HIF-1alpha protein levels, RSUME increases HIF-1 transcriptional activity and induces HIF-1 target genes, like VEGF [1], [9], [13]. RSUME also acts on the proinflamatory transcription factor NF-κB. In contrast to its action on HIF-1, RSUME inhibits NF-κB transcriptional activity [1], and consequently reduces the expression of its target genes, like interleukin-8 (IL-8) [14]. Improving our knowledge on the structure and molecular biology of RSUME isoforms may be relevant for the treatment of pathologies in which SUMO, HIF-1 and NF-κB play essential roles, as well as for the diagnosis and treatment of certain kind of human brain tumors, in which RSUME is expressed, like pituitary adenomas and gliomas [1], [9], [13].

## Results

### The Human RSUME Splice Variants

**Figure 1:**
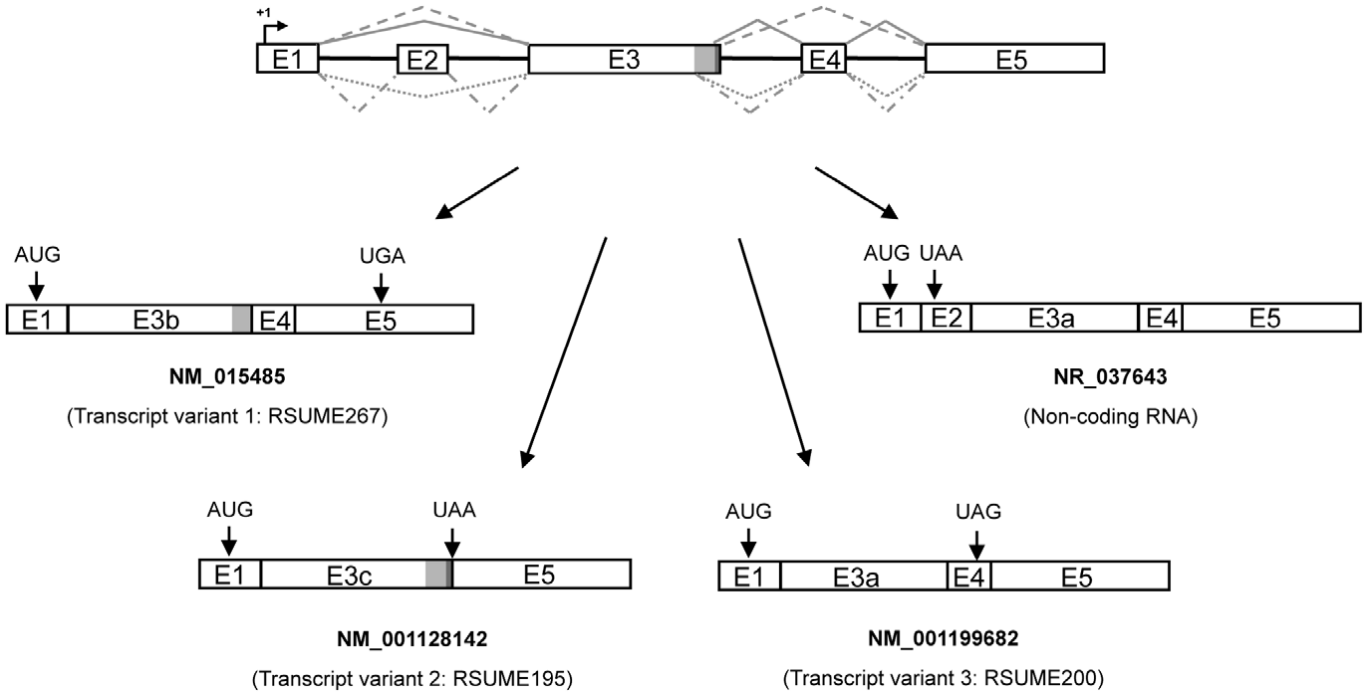
The human RSUME splice variants. Schematic illustration of the RSUME pre-mRNA (above) and the four human RSUME mRNA transcripts splice variants documented to the date. The variant 1 (NM_015485) encodes the longest isoform of RSUME containing 267 aminoacids (RSUME267); the variant 2 (NM_ 001128142) encodes the shortest isoform of RSUME containing 195 aminoacids (RSUME195); the variant 3 (NM-001199682) encodes a 200 aminoacids RSUME (RSUME200); and the variant 4 (NR_037643) is a non-coding RNA because it presents a premature stop codon in the exon 2 that goes to degradation via NMD. Boxes, exons with the exon number inside; filled black lines, introns; gray lines, splicing events (filled, splice event that originates the variant 1; dashed, variant 2; dotted, variant 3; streak and point, variant 4); +1, transcription start site; AUG, translation start site; UAA, UGA and UAG, translation stop sites. This information was obtained from published sequences in the University of California, Santa Cruz, genome browser (http://genome.ucsc.edu/).

Four coding human RSUME mRNA transcripts have been described to date (based on RefSeq database), named RWDD3 (from RWD-domain containing 3) transcript variant 1, 2 and 3, and non-coding RNA. While the variant 1 (NM_015485) has 4 out of 5 exons and encodes the longest RSUME isoform, the transcript variant 2 (NM_001128142) has the longest exon 3, lacks exons 2 and 4, and encodes the shortest isoform. The variant 3 (NM_001199682) does not include exon 2 and has the shortest exon 3. The RSUME mRNA transcripts variants 1, 2 and 3 codify for RSUME isoforms of 267, 195 and 200 aminoacids, named RSUME267, RSUME195 and RSUME200, respectively (Fig. 1). The non-coding RNA (NR_037643) contains all the exons but presents a premature stop codon in the exon 2 that leads it to degradation via NMD. Because of this, such variant does not encode any protein.

### Structural Features of RSUME

**Figure 2:**
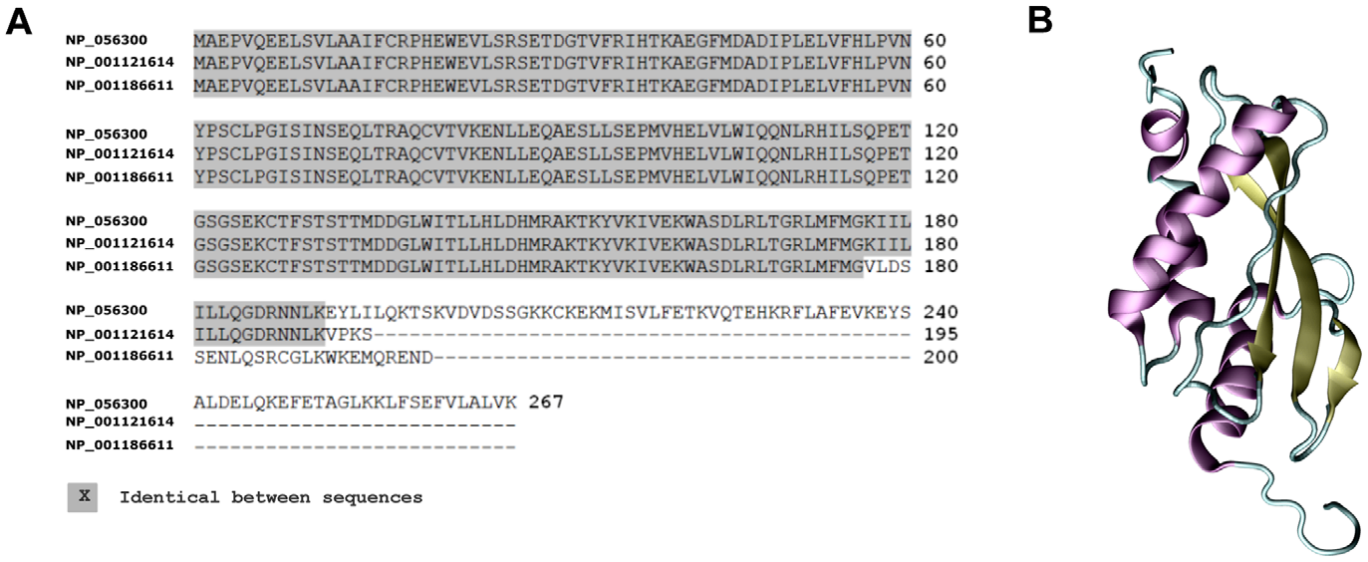
RSUME structural features. **A.** BLAST multiple sequence alignment for RSUME267 (NP_056300), RSUME195 (NP_001121614) and RSUME200 (NP_001186611) isoforms. The identical aminoacids in the alignment are showed in gray. **B.** Ribbon Representation showing the secondary structure elements (alpha helixes are purple, beta-sheets are yellow and loops are cyan) of the RWD domain of RSUME obtained from the PDB (PDBid: 2EBK). The figure was made with the program VMD [29].

The first N-terminal 176 aminoacids are identical in all RSUME isoforms (Fig. 2A). Indeed, with the only exception of its 4 C-terminal residues, RSUME195 is almost contained into RSUME267 and only the 29 C-terminal residues of RSUME200 are exclusive of this isoform. Thus, although all RSUME isoforms share the first 176 residues, they all differ on their C-terminal end.

The best characterized domain of RSUME is the RWD domain, which is required for its SUMOylation enhancement activity, as previously demonstrated by a RSUME mutant carrying two RWD-disrupting point mutations in the RSUME195 RWD domain (Y61A/Y62A) [1]. This domain encompasses aminoacids 1 to 128 and its structure has been solved and deposited in the Protein Data Bank (PDB) by the structural genomics consortium (PDBid: 2EBK) [15] (Fig. 2B). As depicted in Figure S1, the RWD domain consists of three anti parallel beta-sheets and four alpha-helices, and according to the NMR results, the whole domain is flexible, the two central beta-sheets that include residues 39–48 and 56–64 are well defined, the regions spanning residues 31 to 35 and 64 to 83 have a characteristic beta-sheet conformation, and the last region of the domain is a long helix with a kink at residue 116 leading to the formation of two helices. The RWD domain is contained in the N-terminal end of all the RSUME variants (Fig. 2A).

**Figure 3:**
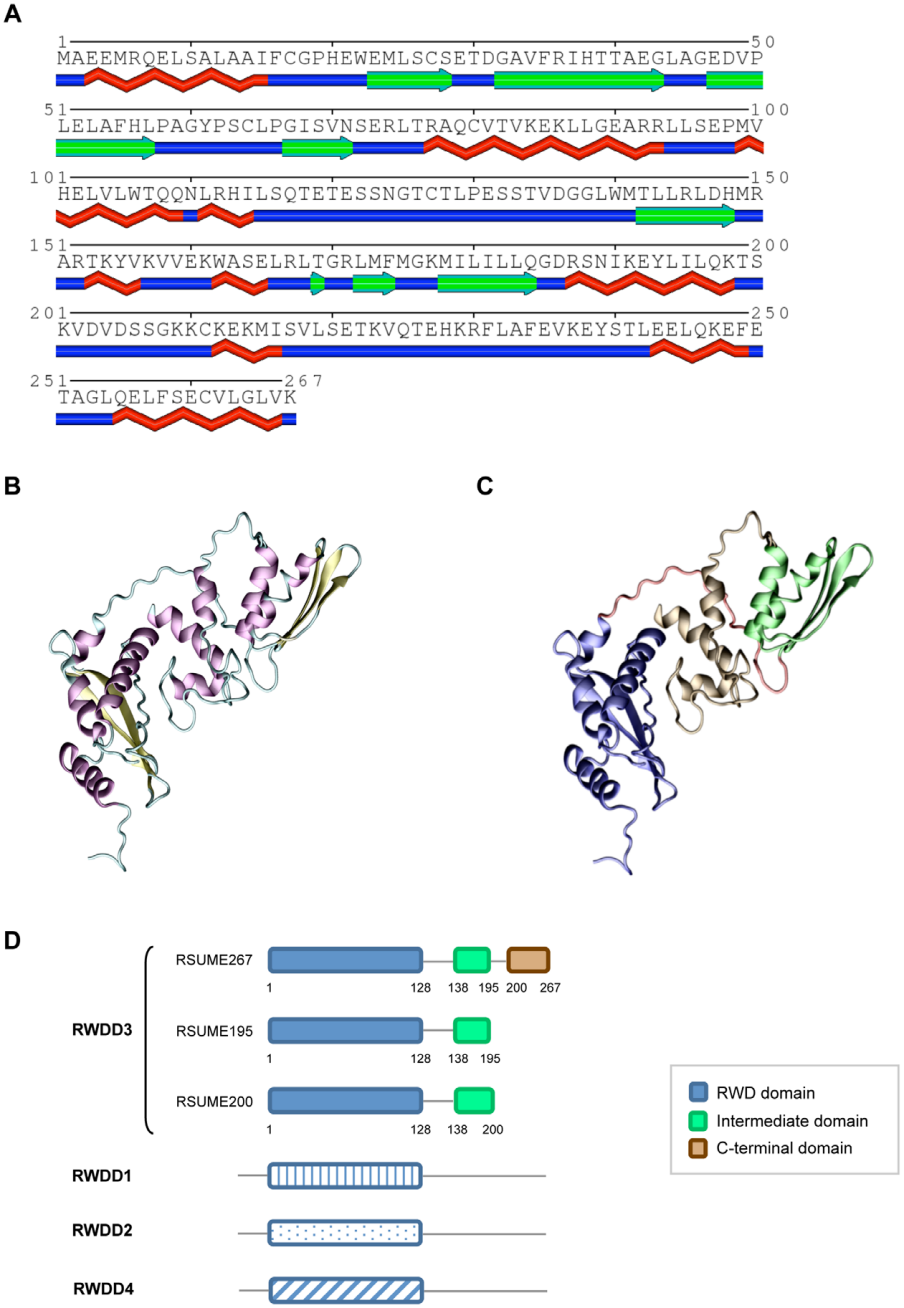
Domain characterization of RSUME. **A.** Secondary structure prediction with PHYRE Server of the whole RSUME267 amino acid sequence. **B.** Ribbon representation of the RSUME267 structure as predicted by the PHYRE Server, showing the secondary structure elements: alpha helixes (purple), beta-sheets (yellow), loops (cyan). The Figure was made with the program VMD [29]. **C.** The same structure that in **B** but different colors encompassing the different regions of the RSUME267 protein. Blue residues 1 to 123, red residues 124 to 137, green residues 137 to 195, brown residues 196 to 267. **D.** Schematic comparison of the domains present in the three RSUME isoforms and in the RWD domain containing superfamily. Boxes, structural domains; lines, loops or unstructured regions; numbers, amino acid position.

The structure and function of the RSUME267 C-terminal half, which spans from residues 128 to 267, have not been characterized to date. In order to study the structural properties of the RSUME C-terminal, we performed secondary structure prediction with the PSIPRED server and disorder prediction with the DISPRO server [16], [17]. We found that there is one region with high probability of being unstructured or disordered, aminoacids 123 to 133. In agreement with this, the secondary structure prediction proposed a loop for this region, suggesting that this loop may be a linker between two domains. Importantly, we also found that the region spanning aminoacids 135 to 267 shows a good secondary structure prediction, suggesting the presence of at least another structural domain in this part of RSUME267 (intermediate domain and C-terminal domain). Taking this information into account we used the PHYRE server [18] for the prediction of the full length RSUME267 structure (Fig. 3A). The PHYRE server allows identifying remotely homologous structures when low sequence identity is found against known structures by using profiles generated by PSI-Blast for both the query sequence and the sequences of known structures. A good secondary structure prediction and a compact domain is predicted for residues 138 to 195, confirming the presence of a second domain in this part of RSUME267 (Fig. 3B and C). This domain is completely included in the RSUME195 and 267 isoforms. Given that the last 24 aminoacids differ in the RSUME200 isoform, we used the same tools to predict its secondary structure. The intermediate domain in RSUME200 is 62 aminoacids long, spans from residues 138 to 200 (the first 38 aminoacids identical in the three isoforms and the last 24 different in the RSUME200 isoform), and has the same folding that in RSUME 267 (a beta sheet followed by a helix, then two beta sheets and then a helix). This result may indicate that the three isoforms have the same fold in their intermediate domains.

Although the confident prediction for the last 67 amino acids of RSUME267 is low to consider it a good prediction, a low disorder prediction together with a predicted tendency to form alpha-helices in the C-terminal last 27 aminoacids suggest that this region is not unstructured. Therefore, we hypothesized that RSUME267 contains an additional domain in its C-terminal end, which is composed by at least two consecutive alpha-helices. Importantly, this domain is present in the longest RSUME isoform, RSUME267, but is absent in RSUME195 or 200, and constitutes the most pronounced structural difference among the C-terminal half of the three human RSUME isoforms. In a BLASTp search [19] in the PDB database with the C-terminal half, we found low identity with all the proteins crystallized so far, including others members of the RWD domain protein superfamily (RWDD1, RWDD2 and RWDD4), which implies that the C-terminal domain is exclusive of the RSUME267 isoform of the RWDD3 family (Fig. 3D). The three RSUME267 structural domains predicted by the PHYRE server are shown in Figure 3C and D, and the proposed C-terminal domain is colored in brown in Figure 3C. Taking into account the difference in the C-terminal RSUME267 and the predicted similarities in the intermediate domain between the three isoforms, we decided to compare RSUME267 with the previous described functions of RSUME195.

### Functional Analyses of RSUME195 and RSUME267 on the SUMO Pathway

**Figure 4:**
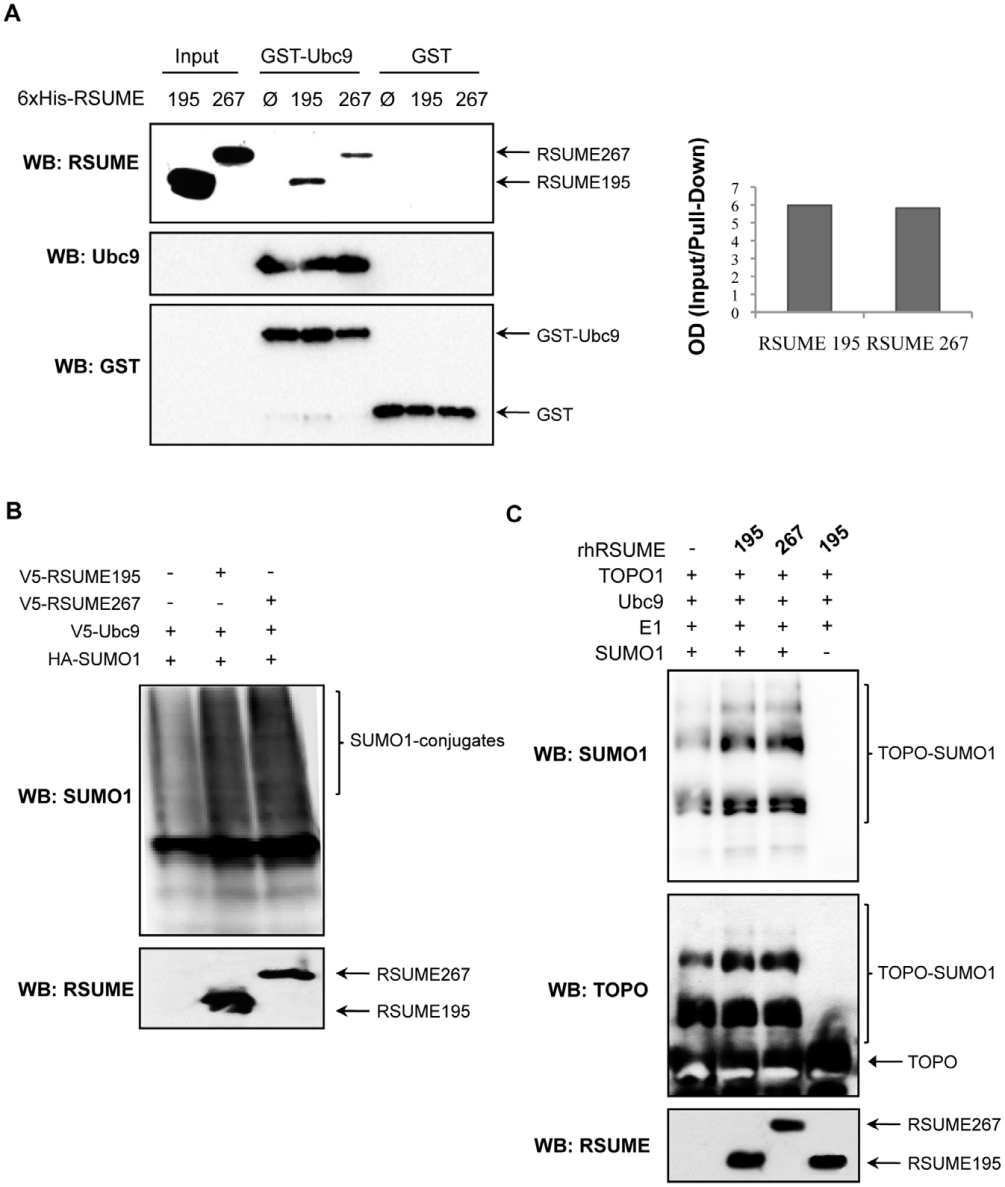
RSUME267 and RSUME195 similar actions on the SUMO pathway. **A.** 1 µg of recombinant RSUME195 or RSUME267 was co-precipitated by GST-Ubc9 in vitro. Pull down experiments were performed and the samples were subjected to Western blot. To compare, the ratio between input and pull-down intensities measured by Image-J Software was calculated. As internal control, to detect unspecific binding, GST alone was used to pull-down any RSUME isoform. Ø, control elution extract purified from *E.coli* transformed with the empty pQE30 vector. **B.** 300 ng HA-SUMO-1, expression vector was co-transfected with 500 ng of V5-RSUME195 or V5-RSUME267 and V5-Ubc9 expression vectors. 48 h post-transfection, cell extracts were subjected to western blot with anti-HA to detect sumoylated proteins. RSUME expression was confirmed with anti-RSUME antibodies. **C.** Sumoylation assay of Topoisomerase I (TOPOI) to test enhancer properties of RSUME isoforms. Experiments were performed in triplicates.

In order to study the functional contribution of the RSUME267 C-terminal domain to the SUMO enhancer properties of this protein, we tested and compared the ability of RSUME195 and 267 isoforms for Ubc9 binding and SUMO-attachment stimulation. As shown in Figure 4A, by *in vitro* pull-down assays, RSUME267 and the shorter isoform bind GST-Ubc9 to similar extents. As an internal control, no unspecific binding was detected when GST alone was used to pull-down any endogenously present RSUME isoforms. We tested the RSUME267 ability to enhance overall protein SUMOylation in cells, and found that this variant increases SUMO conjugation similarly to RSUME195 (Fig. 4B). We also tested the SUMO enhancer properties of this variant on a specific substrate of SUMOylation, and found that RSUME195 and RSUME267 increase SUMO conjugation to Topoisomerase I (TOPOI) to similar extents, as shown in an *in vitro* SUMOylation assay (Fig. 4C). Taken together, these results indicate that these RSUME isoforms do not show differences when evaluating functionally the SUMO pathway.

### Effect of RSUME195 and RSUME267 on NF-κB Pathway

**Figure 5:**
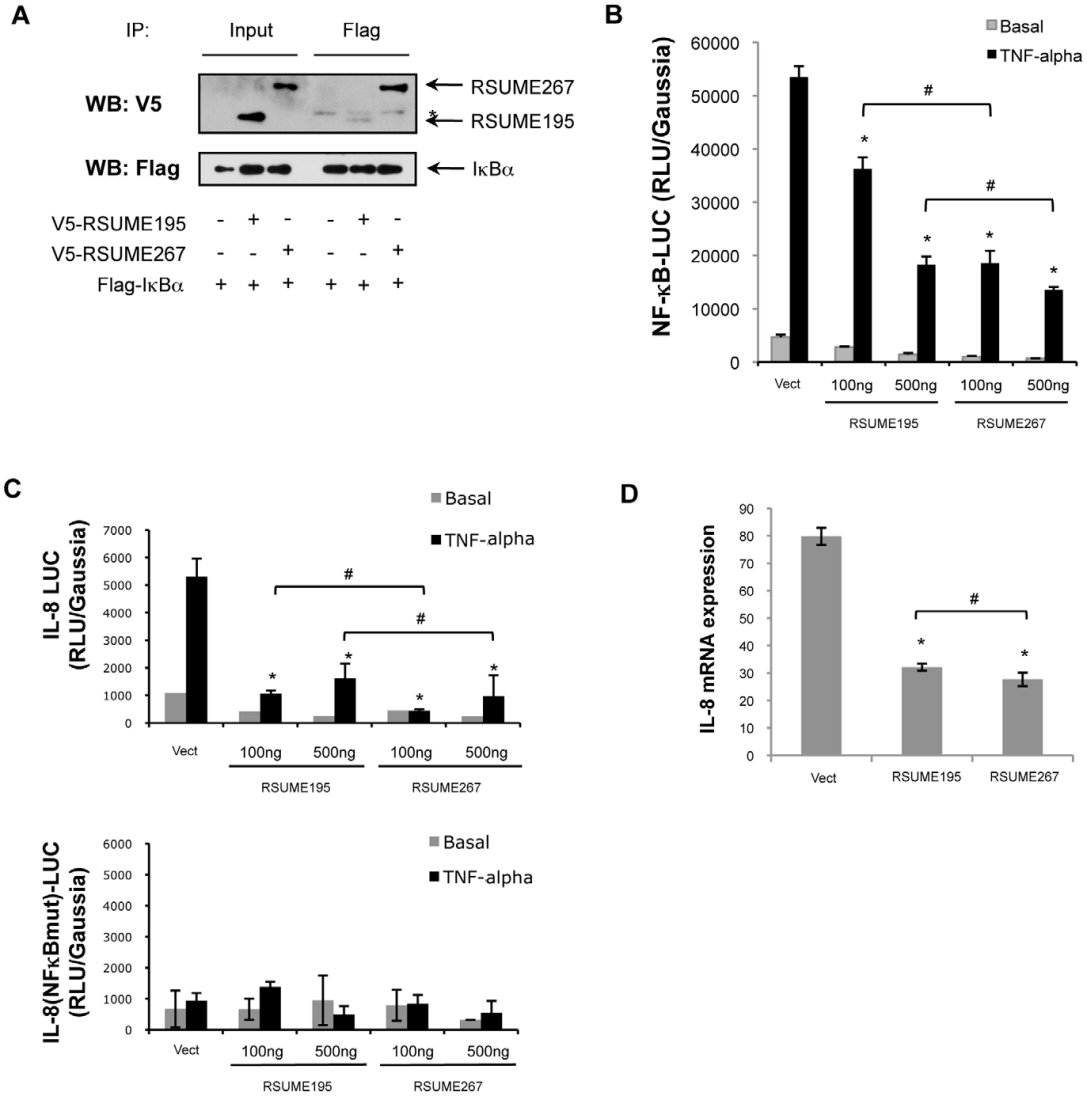
Effect of RSUME195 and RSUME267 over NF-κB signaling pathway. **A.** COS-7 cells were co-transfected with RSUME and IκBα, inmunoprecipitated with anti-Flag antibody, and subjected to western blot with anti-V5 or anti-Flag antibodies. *, band corresponding to an IgG used in the inmunoprecipitation. **B.** and **C.** COS-7 cells were co-transfected with 500 ng of NF-κB-LUC reporter vector (**B**), or IL-8-LUC or IL-8(NF-κBmut)-LUC reporter vector (**C**), 300 ng of Gaussia as control and different concentrations of RSUME195 or 267 vectors (100 or 500 ng). After 24 h cells were stimulated with 10 ng/ml TNF-α for 6 h and luciferase (LUC) activity was measured in the cell extracts. Each value was normalized to Gaussia value. Results are expressed as mean ± SEM from triplicates of one representative experiment of three experiments with similar results. *, p<0.05 compared with cells transfected with the empty vector (Vect) stimulated with TNF-α (ANOVA with Scheffè’s test). ^#^, p<0.05 compared each concentration of cells, stimulated with TNF-α, transfected with RSUME195 vs. RSUME267 (ANOVA with Scheffè’s test). **D.** IL-8 mRNA level was analyzed by quantitative real-time RT-PCR in triplicates in HepG2 cell stimulated with 10 ng/ml TNF-α for 6 h, and the values are given as mean ± SEM after normalization to RPL19. *, p<0.05 compared with cells transfected with the empty vector (Vect) (ANOVA with Scheffè’s test). ^#^, p<0.05 compared the condition transfected with RSUME195 vs. RSUME267 (ANOVA with Scheffè’s test).

In order to study the functional contribution of the C-terminal domain of RSUME267 to NF-κB signaling pathways, we first compared the interaction of the RSUME isoforms with IκBα, a subunit of the NF-κB inhibitor, by inmunoprecipitation experiments. Previously, we have demonstrated a direct interaction of RSUME195 with IκBα. Now, we probe that RSUME267 shows a greater interaction with IκBα than RSUME195 (Fig. 5A). Based on this result, we expect that RSUME267 has a greater impact on the inhibition of the NF-κB transcriptional activity. COS-7 cells were co-transfected with NF-κB-LUC reporter plasmid and different concentrations of RSUME195 or RSUME267 expression vectors. As shown in Figure 5B, although both RSUME isoforms inhibit NF-κB transcriptional activity, low doses of RSUME267 are sufficient for a strong inhibition (65%); 5-folds higher amounts of RSUME195 expression vectors are needed to reach a similar effect (100 ng of RSUME267 vs. 500 ng of RSUME195). Additionally, RSUME267 also has a stronger inhibitory effect on IL-8 promoter activity (Fig. 5C, upper panel). This action of RSUME is absent when the NF-κB binding site of the promoter of IL-8 was mutated (Fig. 5C, lower panel). In addition, we show by quantitative real time RT-PCR that RSUME195 decreases the endogenous mRNA expression of IL-8, and this effect is significantly higher for the RSUME267 isoform, although this effect is very small (Fig. 5D).

Therefore, we conclude that RSUME267, through a greater interaction with IκBα, inhibits more efficiently the NF-κB signaling pathway than RSUME195.

### Effect of RSUME195 and RSUME267 on HIF-1 Pathway

**Figure 6:**
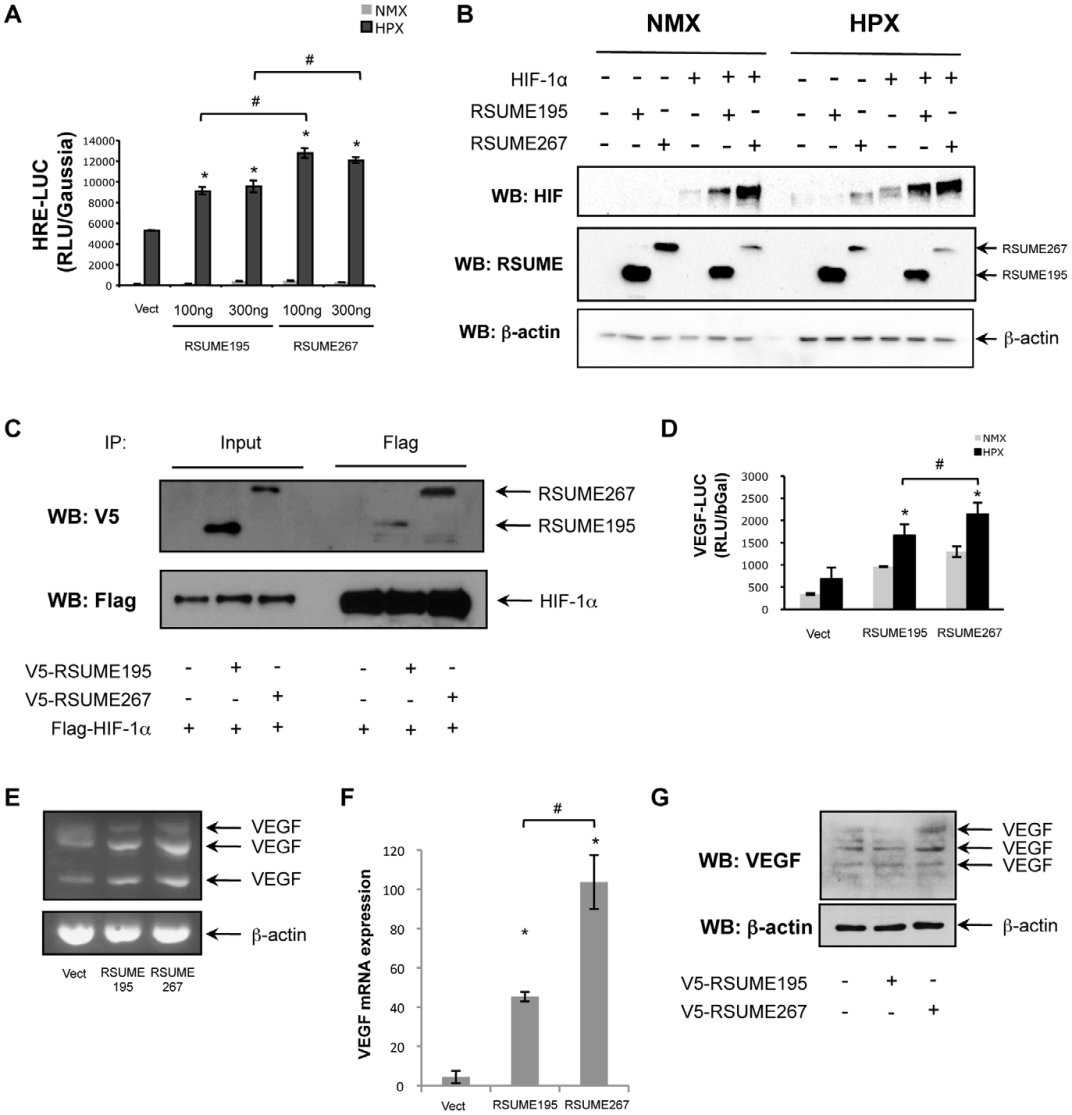
Effect of RSUME195 and RSUME267 over HIF-1 signaling pathway. **A.** COS-7 cells were co-transfected with 500 ng of HRE-LUC report vector, 300 ng of Gaussia report vector and different concentrations of RSUME195 or 267 (100 or 300 ng) to evaluate their effect in HIF-1 transcriptional activity. Twenty-four hours after transfection cells were subjected to hypoxic conditions (1% O_2,_ 5% CO_2_ and 94% N_2_) for 16 h. Then LUC activity was measured in the cell extracts. Each value was normalized to Gaussia value. Results are expressed as mean ± SEM from triplicates of one representative experiment of three experiments with similar results. *, p<0.05 compared with cells transfected with the empty vector (Vect) (ANOVA with Scheffè’s test). ^#^, p<0.05 compared each concentration of cells, under hypoxia, transfected with RSUME195 vs. RSUME267 (ANOVA with Scheffè’s test). NMX, normoxia; HPX, hypoxia. **B.** COS-7 cells were co-transfected with 300 ng of Flag-HIF-1alpha and/or 500 ng of V5-RSUME195 or V5-RSUME267, or the corresponding empty vector (were both are absent). Twenty-four hours post-transfection, cells were subjected to hypoxic conditions (1% O_2,_ 5% CO_2_ and 94% N_2_) for 16 h. Cell extracts were subjected to western blot. NMX, normoxia; HPX, hypoxia; Vect, cells transfected with the corresponding empty vectors. **C.** COS-7 cells were co-transfected with RSUME and HIF-1alpha. Twenty-four hours post-transfection, cells were subjected to hypoxic conditions (1% O_2,_ 5% CO_2_ and 94% N_2_) for 16 h. RIPA cell extracts were inmunoprecipitated with anti-Flag antibody, and subjected to western blot with anti-V5 or anti-Flag antibodies. **D.** COS-7 cells were co-transfected with 500 ng of VEGF-LUC report vector, 300 ng of CMV-β Gal report vector and RSUME195 or 267. Twenty-four hours after transfection cells were subjected to hypoxic conditions (1% O_2,_ 5% CO_2_ and 94% N_2_) for 16 h. Then LUC activity was measured in the cell extracts. Each value was normalized to β-galactosidase value. Results are expressed as mean ± SEM from triplicates of one representative experiment of three experiments with similar results. *, p<0.05 compared with cells transfected with the empty vector (Vect) under hypoxia (ANOVA with Scheffè’s test). ^#^, p<0.05 compared the condition transfected with RSUME195 vs. RSUME267, under hypoxia (ANOVA with Scheffè’s test). NMX, normoxia; HPX, hypoxia. **E.** Semi-quantitative RT-PCR of endogenous VEGF and β-actin mRNA in HepG2 cells transfected with empty vector (Vect), RSUME195 or RSUME267, and subjected to hypoxia (1% O_2,_ 5% CO_2_ and 94% N_2_) for 16 h, twenty-four hours after transfection. **F.** VEGF mRNA level was analyzed by quantitative real-time RT-PCR in triplicates in HeLa cell stimulated with hypoxia for 16 h, and the values are given as mean ± SEM after normalization to RPL19. *, p<0.05 compared with cells transfected with the empty vector (Vect) (ANOVA with Scheffè’s test). ^#^, p<0.05 compared the condition transfected with RSUME195 vs. RSUME267 (ANOVA with Scheffè’s test). **G.** HepG2 cells were transfected with empty vector (Vect), RSUME195 or RSUME267, and subjected to hypoxia (1% O_2,_ 5% CO_2_ and 94% N_2_) for 16 h, twenty-four hours after transfection. VEGF protein levels were analysed by western blot.

Since we have shown that RSUME195 modulates HIF-1 transcriptional activity, we tested if RSUME195 and 267 differ in their stimulatory ability on this signaling pathway. We observed the same result that on the NF-κB pathway (Fig. 6A). RSUME195 and 267 increase HIF-1 transcriptional activity, but lower doses of RSUME267 are necessary to produce the highest effect of the RSUME195 (100 ng vs. 300 ng). To confirm this result, we tested the ability of RSUME267 to increase HIF-1alpha expression. We found that RSUME267 increases HIF-1alpha protein at greater levels than RSUME195 (Fig. 6B). Also, we demonstrate by inmunoprecipitation that RSUME267 has a higher interaction with HIF-1alpha than RSUME195 (Fig. 6C). Analyzing the expression of the HIF-1 target VEGF by a reporter plasmid VEGF-LUC assay, semi-quantitative RT-PCR, quantitative real time RT-PCR and western blot, we conclude that the longest RSUME isoform is more effective in increasing VEGF expression levels (Fig. 6D, 6E, 6F and 6G).

These results, taken together, confirm that the RSUME267 isoform is more potent to activate the HIF-1 signaling pathway due to a stronger interaction with the target HIF-1alpha.

### RSUME195 and RSUME267 Expression

**Figure 7:**
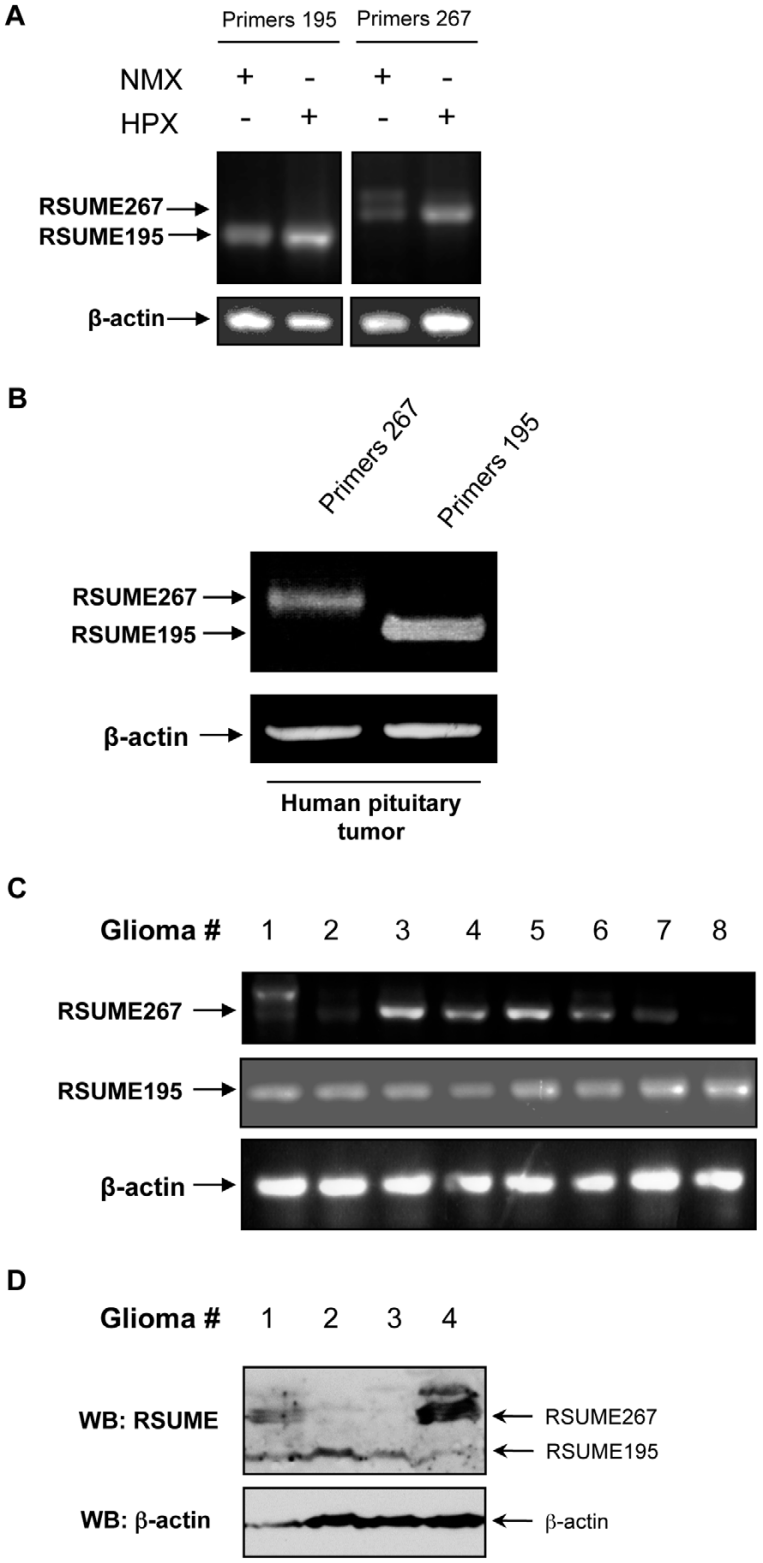
mRNA and protein expression of RSUME195 and RSUME267 in cells and tumors. **A.** Semi-quantitative RT-PCR with specific primers to detect mRNA levels of RSUME195 (left panel) and RSUME267 (right panel), was performed in COS-7 cells exposed to normoxic or hypoxic conditions (1% O_2,_ 5% CO_2_ and 94% N_2_). **B.** Semi-quantitative RT-PCR with specific primers to detect mRNA levels of RSUME195 and RSUME267, was performed on an unstimulated human non-functioning pituitary tumor explant sample. **C.** Semi-quantitative RT-PCR with specific primers to detect mRNA levels of RSUME195 and RSUME267 was performed on human glioma samples. **D.** Western blot analysis of RSUME protein levels was performed on human glioma samples. β-actin protein levels were used as control.

In COS-7 cells and in human pituitary tumors, where RSUME195 has been previously shown to be expressed [1], RSUME267 is also evident in normoxic conditions (Fig. 7A y 7B). In COS-7 cells we also observed co-expression of both isoforms under hypoxic conditions (Fig. 7A). We further evaluated the expression pattern of these RSUME variants in human gliomas, in which RSUME195 expression has been previously shown at protein level [1]. We found low basal level expression of RSUME195 mRNAs in all gliomas analyzed. In contrast, RSUME267 is expressed in most but not in all these tumors (Fig. 7C). Furthermore, RSUME195 mRNA levels are similar in all the tumors tested, but the expression levels of RSUME267 mRNA differ. Similarly, in a second serie of tumor samples, all of the gliomas analyzed showed RSUME195 protein expression, while only a few gliomas showed expression of RSUME267 at protein level (Fig. 7D). These results may suggest nonredundant functions between these two RSUME variants and a constitutive role of RSUME195 in this type of human brain tumors.

## Discussion

We have previously reported that RSUME is a small RWD-domain containing protein with SUMO enhancer properties. In that work, we characterized functionally RSUME195, the shortest RSUME isoform, and showed that this isoform increases protein SUMOylation by interacting physically with Ubc9 and SUMO-1 [1]. Importantly, we demonstrated that RSUME195 action over the SUMO enzymatic cascade depends on the RWD domain, a protein-protein interaction domain [6]. Several RWD-domain containing proteins have been described to date, and the solution structure of most of them has been determined (PDBid: 2EBM,2DAY, 2DAX, 2DAW, 2XZ0) [15]. Interestingly, the RWD domain structure significantly resembles that of E2s ubiquitin-conjugating enzymes [7], [8], [20]. Indeed, it has been demonstrated that the RWD domain is structurally homologous to the E2-SUMO conjugase Ubc9 [7], and based on these data, a structural homology between RSUME and Ubc9 has been proposed [1]. Considering that the RWD domain is contained into all the human RSUME isoforms, an involvement of all these variants on the SUMOylation pathway would be expected. In fact, in this work we show that the shortest and longest RSUME isoforms, which differ in a C-terminal extension of 76 residues exclusive of the longest isoform, increase protein SUMOylation to similar extents. The fact that RSUME195 and 267 actions on overall protein SUMOylation are identical indicates that the C-terminal end of RSUME267 is dispensable for the SUMO-conjugation enhancer properties of RSUME (Fig. 4). The essential role of the RWD domain, as was revealed by RWD-disrupting mutations in RSUME195 [1], supports this idea.

A biological role of the C-terminal end of RSUME267 is suggested by the presence of a defined domain into this region, the C-terminal domain. This domain is contained into the last 67 amino acids of RSUME267, and is composed by at least two consecutive alpha helices spanning on the last 27 residues of this protein. Since this domain is only present in the longest RSUME isoform, we hypothesized that this C-terminal domain could be directly involved in physical and/or functional interactions with a defined subset of RSUME-interacting proteins. We have shown that RSUME195 interacts physically not only with proteins implicated in enzymatic pathways, like Ubc9, but also with substrates of SUMOylation, like IκB and HIF-1alpha [1]. The intrinsic ability of Ubc9 to form homodimers [21] and the structural homology between RSUME195 and Ubc9 support the notion that the nature of these interactions is quite different; while the RSUME-Ubc9 physical interaction would occurs in a RWD-domain dependent manner, SUMO substrates would bind through the C-terminal region of RSUME. Thus, it would be expected that RSUME267 binds preferentially a subset of SUMO targets through its C-terminal domain to favor their SUMOylation. Indeed, we show that RSUME267 has a differential interaction with IκBα and HIF-1alpha.

The proposed structure of the full length RSUME267 protein shows that the RWD domain is separated by a long flexible loop, aminoacids 123 to 133, from its intermediate domain, and the C-terminal domain is located between these two domains with a large interaction surface with both of them. We hypothesize that in the albescence of the C-terminal domain, in isoforms RSUME195 and 200, these two domains may not interact and behave as independent entities. On the other hand, in RSUME267, the C-terminal domain may act as a scaffold producing a more compact 3D structure therefore regulating RWD domain functions, in particular its protein-protein interactions. This is in agreement with our experiments showing differences in the interactions of RSUME267 and RSUME195 with IκBα and HIF1-alpha.

In order to test RSUME functional activity, we first evaluated RSUME267 action over the NF-κB signaling pathway. Desterro and colleagues demonstrated that IκB SUMOylation by SUMO1 leads to its stabilization and consequently inhibits NF-κB transcriptional activity [22]. In agreement with this, RSUME195 interacts physically with IκB, increases its SUMOylation and inhibits NF-κB activity [1]. In this work we found a 5-fold higher inhibition of NF-κB activity by RSUME267 compared to RSUME195. The stronger inhibition on the NF-κB signaling pathway by the longest RSUME isoform suggest that the exclusive C-terminal domain of RSUME267 could belong to the RSUME surface that binds to IκB. RSUME267 binds IκB to increase its SUMOylation, leading to an increased IκB stability and, consequently, to a stronger inhibition of NF-κB transcriptional activity.

Also, we found a more pronounced effect of RSUME267 not only over HIF-1 transcriptional activity but also on HIF-1alpha protein levels and VEGF expression. HIF-1alpha protein levels positively correlate with HIF-1 transcriptional activity. Therefore, it is expected that the higher effect of RSUME 267 on HIF-1alpha protein levels leads to an increase on HIF-1 transcriptional activity and expression of its target genes. In previous works we demonstrated that RSUME195 interacts physically with and increases HIF-1alpha SUMOylation. Thus, as for the case of IκB, the different abilities of RSUME isoforms to modulate the HIF signaling pathway could be explained by the stronger capacity of RSUME267 to interact with HIF-1alpha.

An alternative explanation for the functional differences seen on the RSUME isoforms could be a differential action over E3 SUMO ligases [23]. Although Ubc9 is sufficient for *in *vitro SUMOylation, the action of E3 SUMO ligases significantly increases substrate specificity as well as SUMOylation efficiency *in vitro* and *in vivo* [24], [25], [26]. In agreement with this hypothesis, we have previously shown a synergistic effect between RSUME195 and the E3 SUMO ligase PIAS1 on overall SUMOylation, revealing a functional interaction between these two types of proteins [1]. Thus, the C-terminal domain of RSUME267 could interact preferentially with certain E3s SUMO ligases to promote the SUMOylation of their respective substrate.

Despite the differential action of the different RSUME isoforms on HIF, RSUME195 and 267 are co-expressed in normoxia and are both induced by hypoxia. However, their mRNA and protein levels differ in human glioma cells; while RSUME195 is expressed in all the tumors assayed, RSUME267 is differentially expressed in most but not all.

This work addresses the first open question of RSUME isoforms biology; sharing a degree of functional redundancy among the RSUME isoforms on certain signaling and enzymatic pathways. However, the increased inhibition conferred by RSUME267 over the NF-κB signaling pathway, the increased activation over the HIF-1 pathway and the different expression of the RSUME isoforms suggest specific roles of each RSUME isoform. Improving our current knowledge on the molecular biology of each RSUME isoform will allow us to understand the contribution of each of them to the development and maintenance of certain types of brain tumors, in which RSUME are expressed.

## Materials and Methods

### Computational Methods

To analyze the structural characteristic of the three isoforms of RSUME we performed blastp over the pdb database [19] with each isoform. With isoform b we obtained high similarity with the structure that corresponds to the first 128 aminoacids, (PDBid: 2EBK [15]). No significant similarity was obtained in the other regions of the protein. Secondary structure analysis of the other two isoforms was done with the PSIPRED server and the DISPRO disorder prediction server with the defaults parameters [16], [17]. For modeling of the structure of the full length RSUME protein the PHYRE server [18] was used. The final structures were evaluated using WHAT_CHECK [27]. Structural alignment and visualization were done with the program VMD [28], [29]. Images were rendered with Tachyon [30]. To obtain the domain topology we download the schematic representation used by the PDBsum server [31].

### Cell Line Cultures

COS-7, HepG2 and HeLa cell lines were obtained from the American Type Culture Collection (ATCC, Manassas, VA). Unless otherwise stated, cell culture and reagents were obtained from Invitrogen (Carlsbad, CA), Sigma Chemical Co. (St. Louis, MO), and MERCK (Whitehouse Station, NJ). Cells were cultured in Dulbecco’s modified Eagle’s medium (DMEM) (pH 7.3) supplemented with 10% FCS, 2.2 g/liter NaHCO_3_, 10 mM HEPES, 4 mM L-glutamine, 100 U/ml penicillin, and 100 mg/ml streptomycin.

For NF-κB-LUC, IL-8-LUC and quantitative real time RT-PCR of IL-8 experiments, cells were treated with 10 ng/ml TNF-α for 6 h (R&D, Wiesbaden-Nordenstadt, Germany). For hypoxia, cells were incubated in 2% serum at 37°C, 5% CO_2_, and 1% O_2_ balanced with N_2_ using a hypoxic chamber ProOx Model 110 (BioSpherix, Lacona, NY).

### Tumors

The human pituitary adenoma explant was provided by Dr. A. Carrizo (Neurosurgery section, Hospital Italiano, Argentina) and the human gliomas by Dr. G. Sevlever (Neuropathology and Molecular Biology Department, FLENI, Argentina). This study complies with the June 1964 Declaration of Helsinki, has been approved by the FLENI Biomedical Research ethics committee “Comité de Etica en Investigaciones Biomédicas de FLENI (Fundación para la Lucha contra Enfermedades Neurológicas de la Infancia)”, and informed written consent was received from each patient whose tumor tissue was used in the study. Tumors were shock frozen as soon as possible (10–20 min after surgery), stored at −80°C and later used for mRNA and protein isolation for RT-PCR and western blot assays.

### Plasmids and Transfection Assays

The plasmid constructs were kindly provided and previously described as follows: (NF-κB)3-LUC by Dr. M. Bell [32]; V5-hUbc9, HA-SUMO-1 and Flag- IκBα by Dr. R. Hay [22], [33], [34]; HIF-1alpha expression vector by Dr. M. O. Lee [35]; pBI-GLV4R HRE-reporter plasmid by Dr. E. Van Meir [36]; IL8-LUC and IL8-LUC(NF-κBmut) by Dr. N. Mukaida [37]; VEGF-LUC by Dra. P. A. D’Amore [38]; CMV- β-galactosidase by Dr. D. Spengler [39]; Gaussia-KDEL by Dr. J. Schülke. V5-RSUME195, pQE30-RSUME195, pQE30-SUMO1 and pGEX4-Ubc9 was obtained as previously described [1].

For V5-RSUME267, hRSUME267 was PCR-amplified with a lower primer that included the V5-tag coding nucleotide sequence and was incorporated 3` of the RSUME267 gene. The amplified product was cloned into the pCEFL-HA expression vector kindly provided by Dr. O. Coso [40] in the BamH I/EcoR V sites, which removes the HA tag. For the construction of pQE31-RSUME267, hRSUME267 was subcloned into the Sph I/Xma I sites of the pQE31 bacterial expression plasmid (QIAGEN, Düsseldorf, Germany). Cloning and mutagenesis were performed with standard PCR-based techniques. The nucleotide sequences of all constructs obtained by PCR were confirmed by DNA sequencing.

Transfection assays with lipofectamine reagent (Invitrogen, Carlsbad, CA) were performed as previously described [41] in 6-well plates. Gaussia-KDEL expression vector (coding for the *Gaussia* luciferase gene under the control of the SV40 promoter) or CMV-β-galactosidase expression vector, were used as second reporter control plasmid. Cells were washed with PBS and extracts were prepared with reporter lysis buffer (Promega, Madison, WI). After treatments, cells were harvested and luciferase activity was measured as previously described [42], [43], using the Luciferase detection kit (Promega, Madison, WI) with a Junior luminometer (Berlthod, Bad Wildbad, Germany). In all cases, cells were cotransfected with the Gaussia-KDEL or CMV-β-galactosidase expression vector, used as control for transfection efficiency to standardize the results. Lysates from the transfections were also analyzed by Western Blot as described below.

### Western Blot Assay

Following the appropriate inductions, cells were washed once with PBS (pH 7.0), lysates were prepared in 2X Laemmly sample buffer, separated in SDS-PAGE gels and transferred to nitrocellulose membrane. The membranes were incubated with the corresponding specific primary antibody, followed by incubation with HRP-conjugated specific secondary antibodies (dilution: 1∶3000) (Bio-Rad Laboratories, Hercules, CA), and detection was performed with the ECL kit according to the manufacturer’s instruction (Pierce Biotechnology, Rockford, IL). Anti-RSUME antibodies were generated as previously described [1]. Additionally, the following antibodies were used: mouse monoclonal anti-SUMO1 (Zymed, Carlsbad, CA); rabbit polyclonal anti-V5 and rabbit polyclonal anti-GST (Abcam, Cambridge, UK); rabbit polyclonal anti-IκBα and mouse monoclonal anti-β-actin (C4) (Santa Cruz Biotechnologies, Heidelberg, Germany); mouse monoclonal anti-HIF1alpha (Affinity BioReagents, Rockford, IL); rabbit polyclonal anti-Topoisomerase I (LAE Biotech International, Rockville, MD); mouse monoclonal anti-flag (Sigma, St. Louis, MO); rabbit polyclonal anti-VEGF (Abcam, Cambridge, UK).

### Inmunoprecipitation

COS-7 cells were co-transfected in 6-well plates with 500 ng V5-RSUME isoform and 500 ng IκBα or HIF-1alpha expression vectors. Cells were subjected to hypoxia for 16 h (only in the case of HIF-1), washed twice with ice-cold PBS, lysed on ice with modified RIPA buffer, and immunoprecipitated with the indicated antibodies as previously described [44]. Western blot analyses were performed with the indicated antibodies.

### RNA Isolation and RT-PCR

RNA was extracted as previously described [44]. Briefly, total RNA was isolated from the cells by guanidinium isothiocyanate, followed by the phenol-chloroform method as previously described [41]. RT of 1 µg RNA was performed with MMLV-RT (Promega, Madison, WI) in the presence of RNAsin RNase inhibitor (Promega, Madison, WI) for 1 h at 37°C, followed by an inactivation step at 95°C for 5 min. With the complementary DNA (cDNA) template obtained, a 30-cycle semi-quantitative PCR was performed with the following specific primers: human RSUME195 upper primer: 5′ ATGGATCCGCCATGGCGGAGCCTGTGCAGGAGGAG 3′; human RSUME195 lower primer: 5′ CGTCTAGAATACTTTTTGGCACCTTGAGGTTGTT 3′; PCR product length of 590 bp. human RSUME267 upper primer: 5′ TATATATATAGGATCCATGGCGGAGCCTGTGCAGGAGGAGCTCTCGGTC 3′; human RSUME267 lower primer: 5′ CGGATATCTTAGTCCAATCCCAGCAAAGGGTTAGGTATTTTTACCAGAGCAAGT 3′; PCR product length of 855 bp. β-actin upper primer: 5′ TGGGCCGCTCTAGGCACCA 3′; β-actin lower primer: 5′ CGGTTGGCCTTAGGGTTCAGGGGGG 3′; PCR product length 245 bp. human VEGF upper primer: 5′ CGAAACCATGAACTTTCTGCTGTC 3′; human VEGF lower primer: 5′ TCACCGCCTCGGCTTGTCACAT 3′ which span exons 1 and 8 of VEGF gene product; PCR products length 452, 584 and 656 bp for VEGF121, VEGF165 and VEGF189 respectively [45]. Amplified products were electrophoresed in 1.5% agarose gel and stained with ethidium bromide.

Quantitative real time RT-PCR was performed with cDNA samples of HepG2 and HeLa cells as templates. The amplification reactions of 40 cycles were carried out with specific primers for human IL-8 (upper: 5′-ATTAGCCACCATCTTACCTCACAGT-3′; lower: 5′- GTGCTTCCACATGTCCTCACA-3′), human VEGF-A (upper: 5′-AGAAGGAGGAGGGCAGAATC-3′; lower: 5′-CATCTTCAAGCCATCCTGTGT-3′) and human RPL19 (upper: 5′- GTGTTTTTCCGGCATCGAGCCC-3′; lower: 5′- CAATGCCAACTCCCGTCAGCAGATC-3′). SYBR Green qPCR amplifications were performed in a Stratagene Mx3000P Real-time PCR, and the data were analysed with MxPro Software for Stratagene. For each sample, the values were normalized by the amount of RPL19. All experiments were carried out in triplicates.

### Preparation of Recombinant Proteins

RSUME195, RSUME267 and SUMO-1 were purified from Escherichia Coli M15(pREP4) cells (QIAGEN, Düsseldorf, Germany) with the Qiaexpress Kit from QIAGEN according to the manufacturer’s instructions as previously described [1]. GST-Ubc9 fusion protein was expressed in Escherichia Coli cells and purified with glutathione-Sepharose 4B beads (Amersham Biosciences, Freiburg, Germany) as specified by the manufacturer. Correct protein expression was confirmed by Western blot.

### GST Pull-Down Assay

This assay was performed as previously described [1]. Briefly, three micrograms of GST or GST fusion proteins immobilized on Sepharose beads were incubated with either 1 µg of the indicated recombinant proteins or COS-7 cell lysates transfected with an RSUME expression vector for 1 h at 4°C in GST-binding buffer (20 mM Tris-HCl [pH 7.4], 100 mM KCl, 2.5 mM CaCl_2_, 2.5 mM MgCl_2_, 1 mM DTT, 0.5% NP-40, 1 mM PMSF). Samples were washed four times in lysis buffer, boiled at 95°C for 5 min in 2X Laemmly sample buffer, and separated by SDS-PAGE.

### In Vitro SUMO Conjugation Assays

SUMO conjugation assays were performed as previously described [1] following the manufacturer’s instructions using the Sumoylation Kit from LAE Biotech International (Rockville, MD). Briefly, assays were carried out in a final volume of 20 µl in reaction buffer containing 20 mM HEPES, 5 mM MgCl_2_, 2 mM ATP, 7.5 µg/ml E1, 50 µg/ml Ubc9, 50 µg/ml SUMO-1, 1.0 µg of TOPO (substrate protein), and 0.5 µg of recombinant RSUME where indicated. Reaction mixes were incubated at 30°C for 1 h and stopped by addition of 20 µl of a 2X Laemmly sample buffer.

### Statistics

Statistics were performed by ANOVA in combination with the Scheffé’s test. Data are shown as mean ± SEM.

## Supporting Information

Figure S1 **RWD domain structure.** Schematic representation of the domain topology as obtained from the PDB sum server. RWD domain consists of three anti parallel beta-sheets (pink arrows) and four alpha-helices (red cylinders).
(TIF)
