# Supplementary figures and images for: In Silico Structural and Functional Characterization of the RSUME Splice Variants
Source: PLoS One. 2013 Feb 28;8(2):e57795. doi: 10.1371/journal.pone.0057795 (PMC3585135; doi:10.1371/journal.pone.0057795)

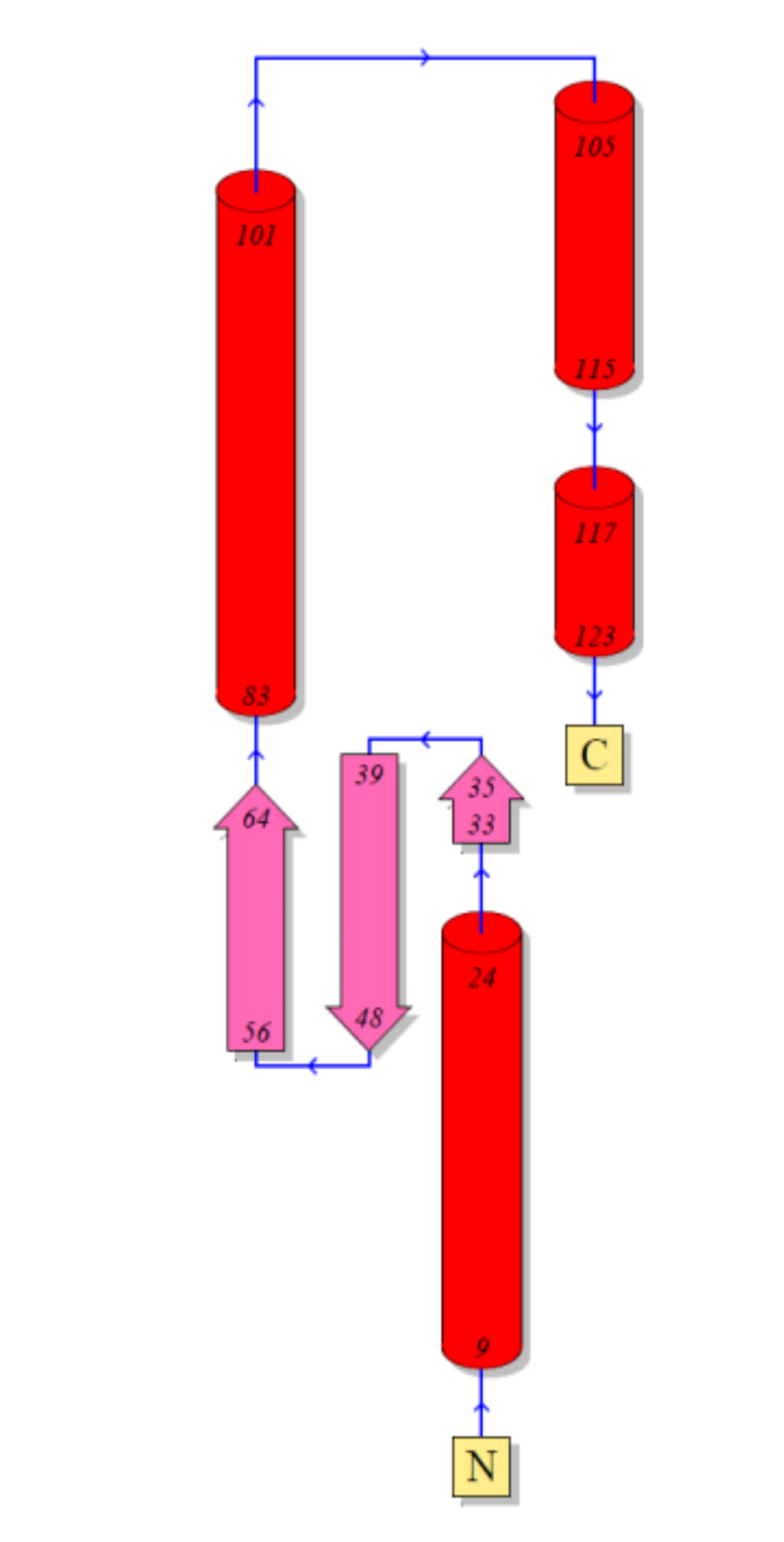

Supplement: Figure S1 — RWD domain structure. Schematic representation of the domain topology as obtained from the PDB sum server. RWD domain consists of three anti parallel beta-sheets (pink arrows) and four alpha-helices (red cylinders). (TIF) [file pone.0057795.s001.tif]
